# '*Stepped procedure*' in laparoscopic cyst decortication during the learning period of laparoscopic surgery: Detailed evaluation of initial experiences

**DOI:** 10.4103/0972-9941.65162

**Published:** 2010

**Authors:** Huri Emre, Akgül Turgay, Ayyildiz Ali, Bağcioğlu Murat, Yücel Özgür, Germiyanoğlu Cankon

**Affiliations:** Department of Second Urology Clinic, Ankara Training and Research Hospital, Ankara, Turkey

**Keywords:** Laparoscopy, renal cyst, *stepped procedure*

## Abstract

**BACKGROUND:**

We evaluated the importance and efficacy of '*stepped procedure*' in laparoscopic cyst decortication as an initial experience in it.

**MATERIALS AND METHODS:**

A 36 renal cyst cases were included. The stepped retroperitonoscopic cyst excision divided into three groups. First step, doing the incisions to place the ports and expanding the retroperitoneal space with balloon distension, second step, placement of trocars and reach to the cyst, third step, aspiration and decortication of the cyst. The difficulty of the sessions was measured with the Visual Analog Scale (VAS) scoring system. Score was determined according to the difficulty of the surgical step ranging from '0' to '10', '0', too easy, '10' too difficult'. The durations were measured. One-way ANOVA test was used for statistical analysis.

**RESULTS:**

The mean age was 52.0 (20-75) years. The mean operation time was 52.0 min. The mean duration of the first step was 12.5, second, 26.0 and third, 22.5 min. The mean VAS of first step, 3.2, second, 6.0 and third, 3.6 There were only significant differences in duration time and VAS score for second step among the surgeons (*P*<0.05).

**CONCLUSIONS:**

Laparoscopic cyst decortication may provide gaining experience to approach the kidney laparoscopically. The side, size and localization of cysts were not found associated with the difficulty of the method.

## INTRODUCTION

Simple renal cysts are common in adults with an incidence of up to 20% at age 40 and 33% at age 60.[1] The increased use of imaging modalities has produced a proportional high degree in detection of disease.[2] The first line of therapy recommended for pain is medical therapy, consisting of anti-inflammatory agents or narcotic analgesics. When medical treatments are ineffective, decompression of cyst is indicated.[3,4] Historically, the options for managing symptomatic renal cysts have consisted of surgical exploration, open decortication or nephrectomy.[5,6] Recently, the use of minimally invasive techniques has become more common to manage renal cysts, including percutaneous aspiration with/without sclerosis, retrograde endoscopic marcupialization guided by flexible ureteroscopy and laparoscopic approach which have been nowadays regarded as the treatment of choice in symptomatic cases with success rate exceeding 90%.[3,7]

The procedure of laparoscopic renal cyst decortication was firstly performed by Hulbert *et al* in the early 1990s.[7] Recently, laparoscopic interventions have gained more popularity as well. Basically, two laparoscopic approaches for cyst decortication were considered as transperitoneal and retroperitoneal approach. This simple procedure could be the first step of laparoscopic surgery during the beginning period for how the urologist should approach the kidney laparoscopically. The learning curve of laparoscopic cyst decortication has never been identified in the literature previously. For this reason, we formed the 'stepped procedure' of laparoscopic cyst decortication at the beginning period. The complexity of the steps has been compared objectively by one surgeon. We report our experience with laparoscopic cyst decortication procedures of two approaches in Bosniak type I renal cysts to evaluate the effectivity of the procedures and the importance of '*stepped procedure*' during the learning period of laparoscopic surgery.

## MATERIALS AND METHODS

Between July 2006 and November 2008, 34 patients with 36 symptomatic renal cysts underwent the laparoscopic cyst decortication in our clinic. In all patients, symptoms were evaluated, physical examination was performed consecutively. The cysts were identified with ultrasonography (USG), enhanced and non-enhanced computed tomography scan (CT scan). Complex cysts, Bosniak type III and IV were not included in the study. Urine analysis, urine culture, count blood cell, serum creatinine and electrolytes were carried out preoperatively. Patients were informed in detail about the laparoscopic surgery, and written informed consent was taken. Retroperitonoscopic cyst decortication was performed to all patients. All procedures were carried out by one surgeon (E.H.) who had finished in a success laparoscopic training course and certified by international applied laparoscopic course, accredited by EBU (European Board of Urology) and Ministry Health of Turkey. However, the surgeon had also trained with laparoscopic training box at least 30 h before start to operate the patients.

***Preparation of retroperitoneal area:*** A 1.5 cm incision below 12^th^ rib in area of Petit triangle was performed. Through the incision, a tunnel was created down to the retroperitoneal space by index finger. A balloon distension system was introduced to create a working space within the retroperitoneum with a visual perception. The balloon was removed afterward 11 mm optic trocar was inserted in retroperitoneal space, and gas insuflation was provided up to 15 mmHg. The other trocars, 5 and 11 mm, were also put with under laparoscopic vision.

***Surgical technique:*** The cystic decortication was performed by using Gyrus^®^ plasmacinetic energy. Following the reach to renal cyst, blunt and sharp dissection was performed. The cyst fluid was aspirated using the suction irrigation device following the cyst excision completed. Internal cyst wall was visualized for if its connection with the renal calices. In the cases of determined connection, double-j stent was inserted. We routinely analyzed the cyst wall pathologically to exclude for unsuspected cystic renal carcinoma. The parameters of cyst laterality, size, localization, operation time and previous surgery were evaluated.

***The aim and identification of 'stepped procedure':*** The aim of dividing the session of retroperitonoscopic cyst decortication into the steps was to determine degree of difficulty for the surgeon who started to laparoscopic surgery as a beginner. The '*stepped laparoscopic procedure'* was divided into three groups. The stepped surgical procedure included the following: first step, doing the incisions to place the ports and expanding the retroperitoneal space with balloon distension, use of laparoscopic equipments; the second step, placement of trocars and reach to the cyst, the preparation of kidney and the third step, aspiration and decortication of the cyst. The difficulty of the sessions was measured by the surgeon (E.H.) with VAS (Visual Analog Scale) scoring system as a difficulty scale following the surgery. Score was determined according to the difficulty of the surgical step ranged from '0' to '10', '0', too easy, '10' too difficult'. At the end of each session, the VAS score of the steps were asked to the primary surgeon. The durations of the steps were measured by assistant surgeon. Patients were discharged when they tolerated an oral diet and their pain was controlled with oral analgesics. All patients underwent radiological follow up with CT scan at 6 months. One–way ANOVA test was used for statistical analysis.

## RESULTS

The characteristics and preoperative parameters of the 36 symptomatic renal cysts in 34 patients, 19 females and 15 males, are shown in Tables 1 and 2. The mean age was 52.0 (20-75). The indication for surgery included right or left loin or abdominal pain in 33; however, one had recurrent haematuria. The mean size of cysts was 7.2 (8-12) cm. Localization of renal cysts was at lower pole in 12 (33.3%), upper pole in 16 (44.4%), middle pole in 8 (22.3%) for 18 (50.0%) right and 18 (50.0%) left kidney. In 2 of 34, applied retroperitoneal approach, cysts were localized in the posterior part of the kidney while the others were located anteriorly. The mean operation time was 52.0 min.

**Table 1:** The general informations of patients

Cases	Age	G*	Time (min)	Laterality	Size (cm.)	PO**	Hematuria	LP***	Local
1	58	F	45	Right	5	+	-	+	Ü
2	52	F	55	Left	5	+	-	+	L
3	43	F	50	Left	9.5-10	-	-	+	M
4	57	F	50	Left	9	-	-	+	Ü
5	20	F	60	Left	6	-	-	+	L
6	69	F	75	Right	8	-	-	+	Ü
7	48	F	75	Right	6	-	-	+	M
8	57	M	65	Right	9	-	-	+	L
9	51	F	57	Right	6	-	+	-	Ü
10	42	M	49	Right	5	-	-	+	L
11	75	M	67	Right	10	-	-	+	M
12	58	F	57	Right	5	-	-	+	Ü/M
13	75	M	48	Right / Left	8/6	-	-	+	Ü/Ü
14	52	M	58	Right / Right	8	-	-	+	L
15	63	F	52	Left	10	+	-	+	L
16	48	F	50	Right	9	-	-	+	M
17	62	M	46	Left	6	-	-	+	M
18	62	M	75	Right	6	-	-	+	L
19	71	M	87	Left	10	+	-	+	Ü
20	53	F	75	Left	5.5-6	-	-	+	Ü
21	39	F	75	Left	7	+	-	+	Ü
22	74	F	44	Right	6	+	-	+	Ü
23	53	F	43	Left	6	+	-	+	M
24	60	F	40	Right	7.5-8	+	-	+	Ü
25	60	F	85	Left	6	-	-	+	L
26	68	M	60	Right	8	-	-	+	Ü
27	49	M	30	Left	5	-	-	-	Ü
28	50	M	50	Right	12	-	-	-	L
29	66	F	35	Left	6	-	-	+	M
30	30	F	38	Left	6.5-7	-	-	+	L
31	68	M	100	Left	6	-	-	+	L
32	67	F	49	Right	5.5-6	-	-	+	M
33	40	M	35	Right	9	-	-	+	Ü
34	66	M	39	Left	9	-	-	+	Ü

* Gender,

** Previous operation,

*** Loin Pain,

F: Female, M: Male, U: Upper, M: Middle, L: Lower

**Table 2:** The demographic features of the patients

Mean age (years)	52.0 (20-75)
Gender (female/male)	19/15
Mean operation time (min)	52.0
Mean cyst size (cm)	7.2 (8-12)
Cyst laterality (right/left kidney)	18 (50.0%)/18(50.0%)

The mean duration of the first step was 12.5, second step, 26.0, third step, 22.5 min [Table 3]. The mean VAS score of the first step, 3.2, second, 6.0 and third, 3.6. The parameters of localization, lateralization and size of the cyst were not significantly associated with the duration time and VAS score for each step (*P*>0.05). The VAS score and duration time of second step were significantly different from the other steps (*P*<0.05). In eight (25.5%) patients, the previous abdominal surgery was detected. All procedures were completed laparoscopically without major complications or conversion to open surgery. Patients were hospitalized for a mean of 1.3 days. None of the patients had urinoma, haematoma and urinary tract infection during the follow-up time. At 6 month, we just detected one recurrence cyst.

**Table 3:** The results of *stepped procedure* of laparoscopic cyst decortication

	Cyst number (n)	Cyst size (cm)	1.step (min)	2.step (min)	3.step (min)	1.step (VAS)	2.step (VAS)	3.step (VAS)
Retroperitoneal	36	7.2	12.5	26.0*	22.5	3.2	6.0*	3.6
approach	(100%)							

* *P*<0.05 Source of support: No support was taken Conflict of Interest: None declared. None

## DISCUSSION

Renal cysts could be congenital or more commonly acquired type. Most patients with simple renal cyst (90-95%) are asymptomatic and most of these cysts are detected incidentally and intervention is not necessary unless it develops symptoms or complications.[8] The most common symptom requiring intervention has been determined as loin pain; another symptoms or complications; hypertension, urinary infection, upper urinary tract obstruction, haematuria and renal failure rarely.[9,10]

The first line treatment of symptomatic simple renal cyst is ultrasound-guided aspiration of the cyst and application of sclerosing agents. This treatment procedure represented a high recurrence rate as well as a risk for accidental spillage of the sclerosing agent which can damage chemically.[11] The open surgical decortication of renal cyst has a high mortality and morbidity. All studies of the laparoscopic approach demonstrated great satisfaction in terms of efficacy, minimal complications, less operative time, minimal blood loss, short hospital stay and well-cosmetic outcome with lower recurrence rate, minimal morbidity and mortality. Laparoscopic decortication of simple renal cysts is a highly effective, safe and minimally invasive alternative to open surgery and antegrade or retrograde endoscopic procedures.[12] The role of laparoscopy in urologic surgery has been increasing in the worldwide. However, there are several obstacles of laparoscopic urological surgery due to the technical difficulties and long learning curve period. Laparoscopic surgery requires a set of skills, instrumentation, optics and depth perception. Laparoscopic surgery in urology differs from its counterparts in general surgery or gynaecology, in that there are no relatively simple procedures suitable for training.[13] However, laparoscopic cyst decortication is the one of the simple procedures and could be performed in the beginning period. Additionally, there has also been increased demand for laparoscopic training courses. In our opinion, following these regular courses, we consider that the laparoscopic cyst excision could be the first option for starting the laparoscopic renal surgery.

The laparoscopic approach to renal cysts has been described via a transperitoneal or retroperitoneal method. Most surgeons prefer a transperitoneal approach in laparoscopic cyst decortication, because they believe that the retroperitoneal approach has limited working space and which can result in difficulty with orientation, visibility, trocar placement and mobilizing the kidney. Recently, the retroperitoneal approach has become more popular. The most important advantage of retroperitoneal approach has been shown that it has a minimal risk of visceral organ injury, urinoma and haematoma confined to retroperitoneum.[8,14,15] Tefekli *et al* reported a radiological success rate of % 88.2 and symptomatic success rate of % 89.5 in a survey on 19 consecutive patients.[16] They preferred the retroperitoneal approach in most of their cases. In another study, minimal morbidity and high effectivity were reported for large cysts of any localization using the transperitoneal approach.[12] In our group, the high rate of previous abdominal surgery did not affect the difficulty of surgery which was approached retroperitoneally. So the bias that could be related to previous surgery was excluded for evaluation of objective parameters in the step of surgery.

We demonstrated that the second step of retroperitoneal cyst excision, placement of trocars, opening Gerota fascia, blunt and sharp dissection to reach the cyst, many technical skills for using laparoscopic instruments (endo-dissector, endo-scissor), was the more difficult and had a longer operation period than the first and third step. These findings were not associated with the side (right or left kidney), localization and size of the cysts. The results confirmed that the laparoscopic cyst decortication could be performed confidentially by the surgeon who just completed the consecutive learning curve stages during the beginning period. The importance of *stepped procedure* in renal cyst decortication could be sought to create a novel learning type for cyst decortication and to investigate the discrepancy of difficulty for various steps. In our opinion, the blunt and sharp dissection with laparoscopic instruments, two-dimensional appearance of endovision and hand coordination be the reasons for longer duration time and high VAS score of second step in overall evaluation. It would be recommended that training in retroperitonoscopic cyst decortication could be focused on blunt and sharp dissection with laparoscopic instruments. The simple attractions of placement trocars and cyst excision following the aspiration were not too difficult to deal with according to the step evaluation.

We suggested that the laparoscopic procedures should be trained steadily not only in advance but also in beginning period. In the literature, the steps of simple renal cyst decortication were not described as we did. So this article is the one which would be the sample for the other laparoscopic urologic surgery for dividing into the steps during the beginning period.

These are the preliminary results for *stepped procedure* in retroperitonoscopic cyst decortication, so the small number of patients could be the restricted factor for the study. However, the surgeon experience during the beginning period was stressed in this study with a novel learning type in retroperitoneal cyst decortication.

## CONCLUSIONS

Laparoscopic cyst decortication could provide gaining experience to approach the kidney. The side, size and localization of the cysts were not associated with the difficulty of the steps in laparoscopic cyst decortication. The second step 'placement of trocars and reach to the cyst, the preparation of kidney' was demonstrated more difficult to do. During the learning period, using the VAS scoring system and the measurement of duration period in surgery and surgical steps to determine the lack of laparoscopic training could be suggested.

## References

[1] Laucks SP, McLachlan MS (1981). Aging and simple cysts of the kidney. Br Radiol.

[2] Hemal AK (2001). Laparoscopic management of renal cystic disease. Urol Clin North Am.

[3] Yoder BM, Wolf JS (2004). Long term outcome of laparoscopic decortication of peripheral and peripelvic renal and adrenal cysts. J Urol.

[4] Shiraishi K, Eguchi S, Mohri J, Kamiryo Y (2006). Laparoscopic decortication of symptomatic simple renal cysts: 10 year experience from one institution. BJU Int.

[5] Holmberg G, Hietala SO (1989). Treatment of simple renal cysts by percutaneous puncture and instillation of bismuthphosphate. Scand J Urol Nephrol.

[6] Stoller ML, Irby PB 3 rd, Osman M, Carroll PR (1993). Laparoscopic marsupialization of a simple renal cyst. J Urol.

[7] Hulbert JC, Hunter D, Young AT, Castaneda-Zuniga W (1988). Percutaneous intrarenal marsupialization of a perirenal cystic collectionendocystolysis. J Urol.

[8] Hemal AK, Aron M, Gupta NP, Seth A, Wadhwa SN (1999). The role of retroperitoneoscopy in the management of renal and adrenal pathology. BJU Int.

[9] Wein AJ, Kavoussi LR, Novick AC (2007). Laparoscopic surgery of the kidney. Campbell-Walsh Urology.

[10] Dunn MD, Clayman RV (2000). Laparoscopic management of renal cystic disease. World J Urol.

[11] Moufid K, Joual A, Debbagh A, el Mrini M (2002). Lumboscopic treatment of simple renal cysts: Initial experience with 17 cases. Prog Urol.

[12] Abbaszadeh S, Taheri S, Nourbala MH (2008). Laparoscopic decortication of symptomatic renal cysts: Experience from a referral center in Iran. Int J Urol.

[13] Keeley FX, Eden CG, Tolley DA, Joyce AD (2007). The British Association of Urological Surgeons: Guidelines for training in laparoscopy. BJU Int.

[14] Hoenig DM, McDougall EM, Shalhav AL, Elbahnasy AM, Clayman RV (1997). Laparoscopic ablation of peripelvic renal cysts. J Urol.

[15] Munch LC, Gill IS, McRoberts JW (1994). Laparoscopic retroperitoneal renal cystectomy. J Urol.

[16] Tefekli A, Altundere F, Baykal M, Sarilar O, Kabay S, Muslumanoglu AY (2006). Retroperitoneal laparoscopic decortication of simple renal cysts using bipolar PlasmaKinetic scissors. Int J Urol.

